# Case of Pulmonary Disease Threatening Phthisis, Relieved by the Supervention of Colica Pictonum, in Consequence of Drinking Cider Impregnated with Lead

**Published:** 1831

**Authors:** John E. Bush

**Affiliations:** Cincinnati


Art. VII.—*Case of Pulmonary disease threatening Phthisis, relieved by the supervention of Colica Pictonum, in consequence of drinking cider impregnated with lead.* By John E. Bush, M. D., of Cincinnati.

I think it unnecessary to offer any apology, for laying the report of the following case before the medical public, when I recollect, that it relates to a disease which humbles the pride of the physician, and generally mocks his best efforts; when I reflect, that in most instances, all that can be done, is to palliate the disease, alleviate the patient's sufferings, smooth the way to, and soften the bed of death. Whatever is calculated to throw any light upon a subject so replete with interest, cannot fail to elicit the attention of every friend of the medical profession, and call forth his most earnest investigation.

The disease alluded to, is Pulmonary Consumption; a disease mostly uncompromising in its characteristics, and irresistible in its progress. Dr. Young says, it carries off, prematurely, one-fourth of the inhabitants of Europe; and M. Baillie will not admit that a genuine case is ever recovered from; at all events, it has been the terror of physicians and the scourge of mankind, from the time of Hyppocrates to the present day.

The subject of the following case is Mr. B—— aged about 26 years, with light blue eyes, thick lips, long neck, sound white teeth, lax fibre, a baker by occupation, temperate as relates to drinking, but careless of his constitution, and somewhat free in his habits in other respects; has lived an athletic life from childhood. On the night of the 22d of February, 1829, his house caught fire, and, extinguishing it, his bed room and bed became very damp, or even wet. He slept in the bed without changing the bedclothes, in consequence of which, he took a violent cold, attended with much cough, slight pain in his breast, and copious expectoration. This continued, with some slight abatement as the warm weather approached, until the 11th of August, when he attended a camp-meeting, and slept in a tent. Whilst there, he was attacked with hæmoptysis, and threw up a large quantity of blood; his expectoration was occasionally tinged with blood, until September, when I was called to see him. When I first saw him, he coughed up, in my presence, at one time, a table spoonful of blood, and muco-purulent matter. At this time he was troubled with fever, alternating with slight rigors every afternoon; night sweats; irritating cough; and pain in his breast, especially when in a recumbent posture; and the disease had all the leading characters of a rapidly progressing consumption. To use the patient's own language, his "cough had always been very loose;" this, taken in connexion with the fact, that the disease, in the first place, showed itself immediately after sleeping in a damp room, (and I ought to have mentioned that he had exerted himself very much in extinguishing the fire, and was in a profuse perspiration when he went to bed,) induced me to consider it, notwithstanding the hæmoptysis which attended it, as a case of the catarrhal variety of consumption.

I bled him about 16 ounces, and directed nauseating doses of ipecac., opium, and calomel. The blood exhibited very slight appearances of inflammation. An antimonial plaster was applied to the chest; the ordinary expectorants were used, such as, squills, antimony, digitalis, sanguinar. canadens., &c. But very little change was effected by this course during the time it was persisted in, which was about three weeks. The cough was sometimes checked for a short period, but soon returned again as bad as ever. After this course was found to fail of success, I directed an emetic of ipecac. to be repeated every two or three days, and in the intervals, the use of opiate expectorants. This course gave some little relief, insomuch that the patient could go about the house every forenoon pretty comfortably; but still had soreness in his breast, troublesome cough, and night sweats. About this time, he was attacked suddenly with pain in his bowels, attended with a sensation of *drawing* towards the navel, incessant vomiting, and obstinate costiveness. On investigating the case, I informed the patient and his friends, that, if it were possible from any exposure to the causes of this disease, I should certainly conclude that he had the *painter's colic*, [colica rhachialgiæ.] but the difficulty was to know how that could have been occasioned. I however gave him pulv. opii. grii. submur hyd. ℈j., and ordered a like dose to be repeated every four or five hours, with the use of enemata, fomentations to the abdomen, &c.; but at my next visit, I found he had obtained little or no relief, I ordered, at the suggestion of my friend, Dr. Finley, the addition of ol. croton tigl. to the calomel and opium. We at this visit were informed that Mr. B. had converted a *soda fount* into a *cider fount*, which had a leaden tube to conduct the cider to the counter, through which he had been in the habit of drinking cider. This at once removed the difficulty of accounting for the disease, and confirmed my first suspicions. By continuing those remedies three or four days, the obstruction of the bowels was partially removed, the vomiting somewhat abated, and the pain very sensibly lessened. By continuing the cathartics and opiates for several days more, the disease of the bowels entirely disappeared. And this brings us to the important item in the case.

When the pain in the bowels and the vomiting were at their height, the pain in the breast, cough, and night sweats entirely disappeared, and all the previous symptoms of pulmonary disease ceased altogether. It was rational, however, to expect that, when the disease of the bowels was removed, the lungs would return to their former condition; but, to my great surprise and gratification, the patient returned to a state of health which has continued to the present time, nearly six months, notwithstanding the winter has been very violent, and the thermometer has two or three times in the course of the winter changed forty degrees in twenty-four hours.

There are several important questions suggested by this case.

1st. Did the restoration of the lungs depend upon any specific effect of the lead?

2dly. Was the vomiting, attendant on the colic, efficacious in removing the pulmonary complaint?

3dly. Was it effected by counter-irritation?

4thly. Did it depend upon the conjunct influence of the whole of them?

These are questions which I think of much importance to the medical profession, and the hope of seeing them satisfactorily answered, has principally induced me to publish this case. If it should, in any way, contribute to the more successful treatment of pulmonary complaints, I shall be abundantly compensated for the little pains I have taken in placing it before the public.

